# Depression increases the onset of cardiovascular disease over and above other determinants in older primary care patients, a cohort study

**DOI:** 10.1186/s12872-015-0036-y

**Published:** 2015-05-12

**Authors:** Harm W. J. van Marwijk, Koen G. van der Kooy, Coen D. A. Stehouwer, Aartjan T. F. Beekman, Hein P. J. van Hout

**Affiliations:** Department of General Practice and Elderly Care Medicine, EMGO+ Institute for Health and Health Care Research, VU University Medical Center, Amsterdam, The Netherlands; Rotterdam Business School, P.O. box 2941, , 1000 SN Rotterdam, The Netherlands; Department of Internal Medicine, Maastricht University Medical Centre, Maastricht, The Netherlands; Department of Psychiatry, EMGO institute for Health and Care Research, VU University medical center and Academic Outpatient Clinic for Affective Disorders, GGZ inGeest, Amsterdam, The Netherlands

**Keywords:** Major depression, Primary care, Elderly, Cardiovascular health

## Abstract

**Background:**

To determine if major depressive disorder (MDD) in older primary care patients is an independent risk factor for cardiovascular events.

**Methods:**

A cohort of 143 primary care patients with depression and 139 non-depressed controls without depression (both aged over 55 years, matched for age and gender) from the Netherlands was evaluated for 2 years. MDD was diagnosed according to DSM-IV–criteria. During the follow-up period, information was collected on physical health, depression status and behavioural risk factors. CVD end points were assessed with validated annual questionnaires and were crosschecked with medical records.

**Results:**

Thirty-four participants experienced a cardiovascular event, of which 71 % were depressed: 27/134 with MDD (20.1 %) and 9/137 controls (6.6 %). MDD was associated with a hazard ratio of 2.83 (*p* value 0,004, 95 % CI 1.32 to 6.05) for cardiovascular events. After adjustment for cardiovascular medication, the hazard ratio was 2.46 (95 % CI 1.14 to 5.30).

**Conclusions:**

In a 2-year follow-up period, baseline MDD increased the risk for CVD in older primary care patients compared with controls, over and above well-known cardiovascular risk factors.

## Background

The World Health Organization (WHO) projects that both cardiovascular disease (CVD) and major depressive disorder (MDD) generate the greatest loss of ‘disability-adjusted life years’ in 2030 [1]. CVD is a leading cause of death worldwide [2]. However, whether MDD is also an independent risk factor for the onset of CVD remains controversial [3–5]. With MDD being a possible modifiable risk factor for CVD, more research is necessary to examine to what extent MDD poses a risk for the onset of CVD and by what mechanism it functions.

Only a few of the studies that take into account a large number of risk factors have examined longitudinal associations between MDD and CVD. Studies with positive associations show much heterogeneity in methodology and outcomes. Two studies found an association but only among elderly women [6, 7]. Ariyo found an increased risk but only for those with the highest mean depression score [8], whereas Penninx found an association for newly depressed but not for chronically depressed patients [9]. MDD is positively associated with excess mortality [5, 10]. Luukinen could only find an association between MDD and subsequent sudden cardiac death [11]. However, in a 12-year prospective cohort study among elderly non-institutionalized Australians, depression was not associated with CVD incidence or mortality [12].

The screening of over 5000 Dutch persons aged 55+ years visiting their general practitioner (GP) showed a high prevalence of 14 % of MDD among this group, with only 23 % being treated with antidepressants [13]. MDD, CVD and their risk factors are all prevalent in older adults. We, therefore, wanted to examine in a prospective 2 year cohort study to what extent older GP patients with MDD indeed have an increased risk of developing CVD, and, if so, whether the increased risk is independent of other known risk factors for cardiovascular events.

## Methods

### Study design

This longitudinal two-year prospective cohort study compared 143 primary care patients with depression and 139 non-depressed controls without depression (both aged over 55 years), on the primary endpoint of CVD morbidity and mortality.

### Setting

This study was conducted in 14 general practices in the Netherlands, from February 2002 to July 2003.

### Participants

The eligibility criteria were that patients fulfilled criteria for MDD according to the Diagnostic and Statistical Manual of mental disorders IV (DSM-IV). The exclusion criteria included severe cognitive impairment (Mini-Mental State Examination score below 18), treatment for current depression or treatment within the past six months (antidepressant therapy or psychotherapy) and treatment for psychosis.

#### Sources and methods of selection

Individuals of 55 years of age or older visiting one of the participating general practices were recruited by means of a two-stage screening procedure as previously described in detail [14]. This two-staged screening was considered the most practical approach. Firstly, individuals were sent a validated depression screening questionnaire (*n* = 6719): the Geriatric Depression Scale 15-item (GDS-15) [15]. Secondly, respondents (*n* = 4301) who scored five points or more (*N* = 843) on the GDS-15 were asked to participate in a diagnostic interview by a trained interviewer using the validated Primary Care Evaluation of Mental Disorders (PRIME-MD, range 0–9) [16]. The participating MDD patients were matched on sex and age to a person with a negative GDS-15 score of less than five. One week prior to the physical examination, the severity of the depressive symptoms was measured with the Montgomery Åsberg Depression Rating Scale (MÅDRS) [17]. Controls with a score of ten or higher (*n* = 11) on the MÅDRS, and MDD patients who scored under 10 (*n* = 8) were excluded from the comparison.

#### Follow-up

During the follow-up period of 24 months, data were collected every 6 months by means of a) telephone interviews for the psychopathological and cardiovascular measures and b) written questionnaires for the lifestyle domains. All participants gave their written informed consent prior to inclusion in the study. The ethical committee of the VU University Medical Centre approved the study.

#### Matching criteria and number of controls per case

Of the 432 potential participants with depression, 300 with depression were expected to be available (20–25 % non-response) for interviews and half was expected to have MDD. The screening and interview procedure resulted in the inclusion of 143 primary care participants with MDD and the exclusion of 62 persons.

### Variables

#### Outcomes

CVD included ischaemic heart disease (acute myocardial infarction, angina pectoris or chronic ischaemic heart disease: ICD-10 code I20-25), cerebrovascular disease (transient ischaemic attack (TIA) or stroke: ICD-10 code I60-69 and G45), and other (heart failure, conduction disorders and vascular disease: I34-37, 45-50, 71-77). A previous history of CVD was assessed by asking the participants if they had ever had a heart attack or other heart condition, vascular disease, TIA or stroke. Independent interviewers assessed CVD events through interviewing the participants and these were crosschecked with the GP medical files to determine the time of the event. All events were coded according to the International Classification of Diseases, Tenth Revision (ICD-10).

#### Exposures

At baseline, a trained independent interviewer used a structured interview and performed physical and laboratory examinations. More details of the methods are described elsewhere [14]. The interview and physical and laboratory examinations covered:

- Demographics
- CVD disease status. This was determined by asking about actual CVD, medical history and the presence of hypertension, hypercholesterolemia and diabetes mellitus.
- Cardiovascular medication was classified according to the Anatomical Therapeutic Chemical classification (ATC code). Use of beta-blockers, calcium antagonists, ACE-inhibitors, diuretics and other medications within the C category of the ATC system was taken as additional evidence of CVD or cardiovascular risk factors (yes/no).
- Lifestyle factors such as smoking (current smoker or non-smoker) and physical inactivity (the modified Baecke questionnaire for elderly) [18].
- Physiological factors such as i) systolic and diastolic blood pressure (SBP, DBP), ii) body mass index (BMI), iii) heart rate variability (HRV) which is the standard deviation of all NN intervals on the electrocardiogram (ECG): HRV SDNN ln. HRV may be a prognostic factor for CVD and be more prevalent among depressed patients [19]; iv) fasting glucose (FG), v) High and Low Density Lipoprotein (HDL and LDL) and vi) inflammation markers such as CRP and interleukin-6 (IL-6) [20–22].
- Psychopathology. To evaluate whether patients fulfilled MDD criteria according to DSM-IV, we used the validated PRIME-MD interview. The severity of observer-rated depressive symptoms at baseline was measured with the MÅDRS. Anxiety was assessed with the anxiety scale of the Hospital Anxiety Depression Scale (HADS-A) [23].

All of the above exposure variables were tested in bivariate association with the onset of CVD among persons with MDD and controls.

### Study size

In a sample of older adults, we assumed to find at least 28 % new cardiovascular events in 3 years in the MDD sample and 14 % in the controls [24]. With α = 0.05 and β = 0.2, and the expected percentage difference for new CVD events of 14 % (28–14 %), a control-group of 145 would provide sufficient power to answer our question.

### Quantitative variables handling

Variables not normally distributed were log-linear transformed (SDNN, triglycerides, CRP, IL_6).

### Statistical methods

To determine whether and to what extent the elderly GP participants with MDD had an increased risk of developing CVD over controls (the primary hypothesis), we first compared baseline variables. Differences in demographic, cardiovascular, psychiatric, lifestyle and physiological factors between depressed and non-depressed subjects were evaluated using Student’s *t*-test for continuous data and Chi-square test for dichotomous data.

To examine the effect of MDD on time to cardiovascular event and adjust for confounding, we computed the amount of cardiovascular events occurring within the follow-up period in MDD participants and controls with Cox regression models. To assess whether potential confounders influenced the risk of CVD associated with depression, we included variables into the model that were significantly different at baseline and had significant bivariate associations with cardiovascular events. Data were analysed using SPSS 20 and two-tailed test α levels of <.05 were used to define statistical significance. We checked effect modification between prior CVD status and depression by adding an interaction term.

## Results

### Participants

**Fig. 1:**
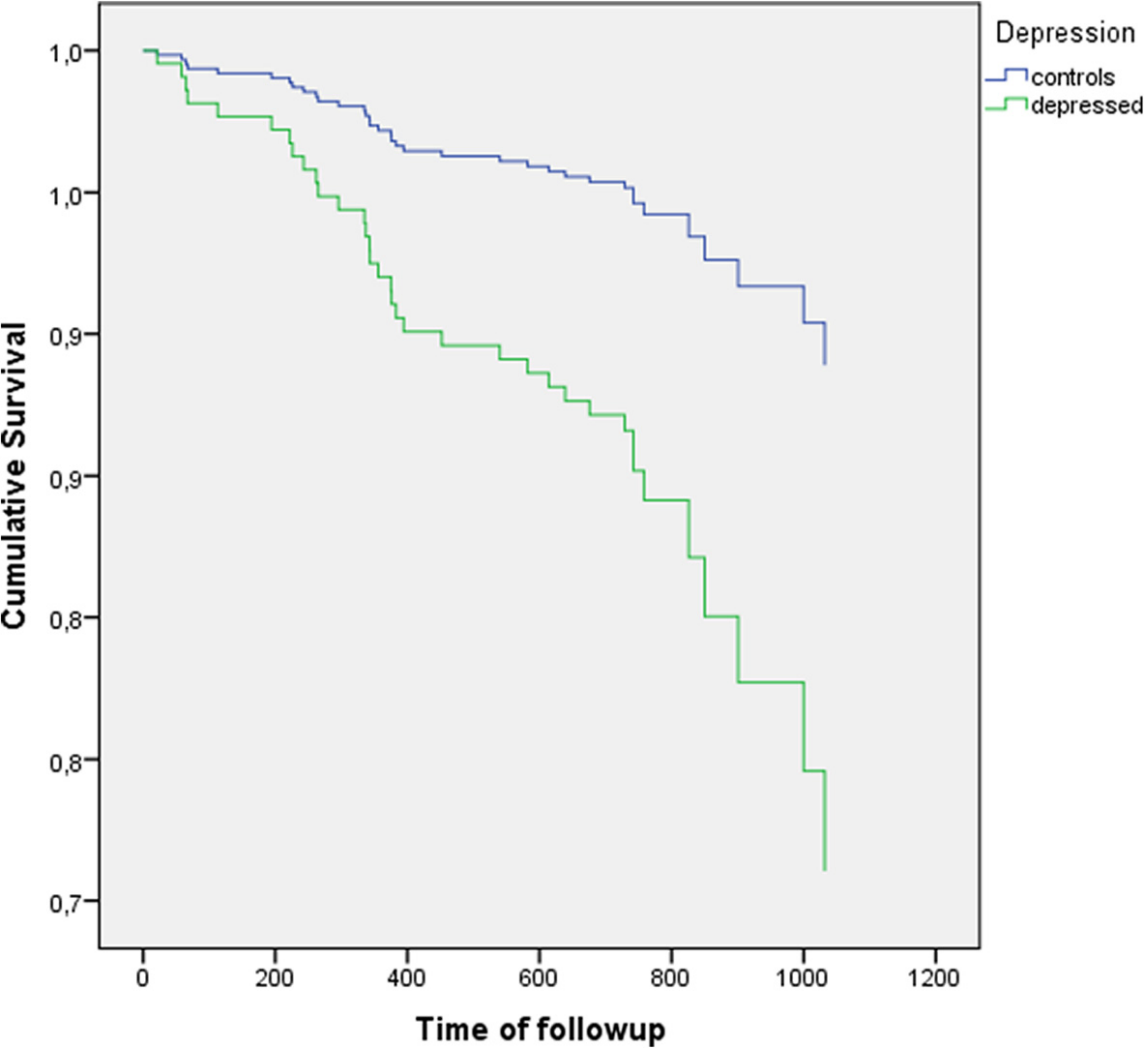
The cumulative survival of controls and depressed cases over a 2 year follow-up

A total of 6719 persons was screened for depressive symptoms using the GDS-15. Of the approached GP patients, 293 were identified to have MDD. Of these patients, 187 were eligible and 76 % of them participated in our study (*n* = 143). The non-participants (ineligible, refused) had a comparable gender distribution (66.7 vs. 64.0 % female), but were older (71.55 vs. 67.59 years) and had a lower average PRIME-MD score (5.86 vs. 6.37) than the 143 participating index participants. Fig. 1 presents a flowchart of the recruitment.

#### Descriptive data

Participant characteristics are briefly presented in Table 1. The matching process was not entirely successful as 99 women (69 %) and 44 men (31 %) were included in the depressed group and 90 women (65 %) and 44 men (35 %) % in the controls (p 0.423). Background characteristics showed that the two groups were comparable, except that participants with MDD lived alone more often than controls (*X*^2^ = 12.44, *p* < 0.001). Both groups had a somewhat increased CVD risk at baseline. The prevalence of diabetes was higher among depressed patients than among controls (*X*^2^ = 5.75, *p* 0.017), but fasting glucose levels did not differ, indicating that the diabetics were properly treated for their condition. Pertaining to lifestyle factors, participants with MDD were physically more inactive as shown by their lower mean Baecke physical activity score (*p* < 0.001). The following physiological factors were different: BMI levels were 28.2 (SD 5.2) kg/m^2^ and 26.8 (SD 4.2), with a statistically significant difference at *p* 0.0018, and the MDD group had a lower mean HRV as measured on an ECG (*p* 0.023). Depressed and non-depressed participants showed no differences in cholesterol levels or other physiological parameters.

**Table 1:** Differences in baseline characteristics between participants (*n*, %)

Category	Risk factor	MDD	Controls	Adjusted HR (95 % CI)
Background	Age, y, mean (SD)	67.9 (8.7)	67.6 (7.5)	
	Gender (male)	44 (30.8)	49 (35.3)	
	Living alone**	72 (50.3)	41 (29.7)	
	Education (low)	55 (38.5)	62 (44.6)	
	Religion (yes)	70 (49.0)	64 (46.0)	
CVD status	CVD*	31 (25.6)	19 (14.1)	
	Hypertension	54 (44.6)	48 (35.6)	
	Hypercholesterolemia	32 (26.4)	31 (23.0)	
	Diabetes*	14 (11.6)	5 (3.7)	
	Family history of CVD	56 (39.2)	56 (40.3)	
	CVD medication**	85 (59.4)	62 (40.6)	2.54 (1.19 to 5.44)
	Chron. illnesses, mean (SD)*	3.9 (2.3)	3.1 (2.1)	
Lifestyle	Smoking current	32 (22.4)	19 (13.7)	
	Baecke, mean (SD)**	5.42 (5.09)	7.86 (5.17)	
Physiology (mean, SD)	BMI, kg/m^2^*	28.17 (5.21)	26.76 (4.16)	
	SBP, mmHg	146.2 (18.05)	142.4 (18.56)	
	DBP, mmHg	83.62 (9.41)	83.72 (8.17)	
	HRV SDNN ln*, ms	1.50 (0.25)	1.57 (0.24)	
	Total cholesterol, mmol/l	5.98 (1.15)	6.02 (1.08)	
	Fasting glucose, mmol/l	5.64 (1.13)	5.56 (0.96)	
	LDL cholesterol, mmol/l	3.54 (1.07)	3.74 (0.94)	
	HDL cholesterol, mmol/l	1.65 (0.51)	1.60 (0.41)	
	Triglycerides, mmol/l	1.76 (1.16)	1.55 (0.92)	
	IL-6 ln, pg/ml	0.95 (0.63)	0.86 (0.62)	
	CRP ln, mg/l	1.02 (1.08)	0.77 (1.08)	
Psychiatry	MADRS, mean (SD) **	19.38 (8.25)	2.73 (3.89)	
	HADS-A, mean (SD) **	9.92 (2.89)	5.98 (2.46)	
	Panic disorder**	36 (25.4)	7 (3.2)	
	Depression history**	115 (81.0)	63 (45.7)	
	Family history depression*	58 (40.6)	39 (28.1)	

*, *p* < 0.05; **, *p* < 0.01

Depressed participants reflected prevalent cases (81.0 % with a history of depression), but many of the controls also had such a history (45.7 %, (*X*^2^ = 37.73, *p* < 0.001). As was to be expected, cases scoring much higher on the MÅDRS (reflecting our case–control design) had greater co-morbid anxiety (average four points higher on HADS-A) (t12.25, df = 279, *p* < 0.001) and were five times more likely to report a panic attack in the last month (*X*^2^ = 22.37, *p* < 0.001) than controls. Anxiety levels of HADS-A of 9.9 (SD 2.9) and 6.0 (2.5) were in the moderately high range.

### Outcome data

During the follow-up period of on average 743 days, 36 cardiovascular events were registered: 27 among the depressed participants and 9 among non-depressed participants (Table 2). Ischaemic heart disease was the cause of 9.0 % (*n* = 13/143) and 0.7 % (*n* = 1/139) of cardiovascular events in the depressed and the non-depressed group, respectively. Cerebrovascular events accounted for 1.4 % of events in the depressed group versus 2.9 % in the non-depressed group. 7.7 % of events in the depressed group were due to other cardiovascular disease, in comparison to 3.6 % in the non-depressed group. Eight participants died during the follow-up period, three of cardiovascular disease (two depressed participants and one non-depressed participant) and one depressed participant of non-cardiovascular disease. Four-deaths were due to unknown reasons, and all occurred in the depressed group.

**Table 2:** CVD events and mortality

Event (ICD-10 code)	MDD	Controls
	(N = 143), %	(n = 139), %
Ischaemic heart disease^a^ (I20-25)	13 (9.0)	1 (0.7)
Other CVD^b^ (I34-37, 45-50, 71-77)	11 (7.7)	5 (3.6)
Cerebrovascular disease^c^ (G45, I60-67)	3 (2.1)	3 (2.2)
No event	107 (74.1)	128 (92.1)
Death with unknown cause (R99)	4 (2.8)	0 (0.0)
Missing data	5 (3.5)	2 (4.4)

^a^Includes angina pectoris, acute myocardial infarction and chronic ischemic disease

^b^Includes conduction disorders, heart failure, nonrheumatic valve disorders and aortic aneurysm/dissection

^c^Includes transient ischaemic attack and stroke (ischemic and non-ischemic)

### Main results

**Fig. 2:**
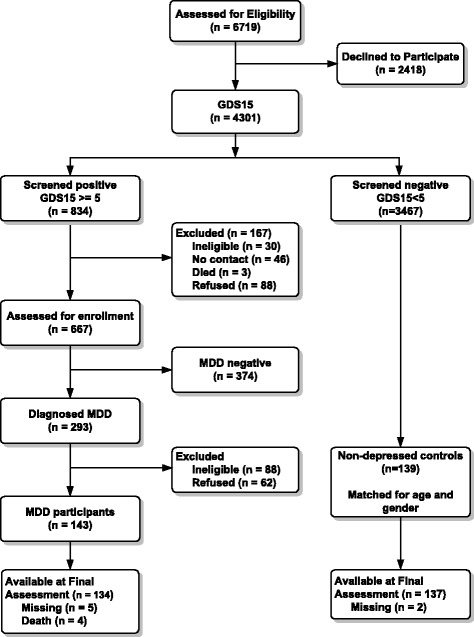
Flow chart of the recruitment of participants

In bivariate analysis between covariates and cardiovascular events in on average 743 days, depression and a history of CVD increased the hazard ratio for cardiovascular events significantly (Table 1). Depression had an unadjusted hazard ratio (HR) for CVD events in older general practice patients of 2.83 (95 % confidence interval (CI) 1.32 to 6.05). As the MADRS score was highly correlated with depression status (Pearson correlation 0.79), we excluded it from the analyses. Only CVD medication at baseline was significantly associated with both baseline depression status and outcome and remained in the model. After adjustment for CVD medication, the hazard ratio decreased slightly to 2.46 (95 % CI 1.14 to 5.30). Other models showed similar slight reductions in HR for depression on cardiovascular events. Survival curves according to the adjusted Cox model are shown in Fig. 2. No evidence for effect modification between prior CVD status and depression was seen.

## Discussion

In this study on the longitudinal association between depression and cardiovascular diseases, we found that older general practice patients with major depressive disorder were significantly more likely to have a cardiovascular event during two years of follow-up than non-depressed elderly participants. CVD medication confounded the association between MDD and CVD events, but other known risk factors, including co-morbid CVD, lifestyle factors and physiological factors did not.

Previous investigations on the association between depression and CVD events have had mixed results [7], or even no result [25]. A recent systematic review of fifteen studies with people without heart disease who were exposed to depression concludes that most but not all studies find an association between depression and cardiac outcome. Our estimate seems higher than their overall relative risk (RR) estimates of approximately 1.5 (0.64 to 6.9) for developing an acute coronary syndrome. Our impression is that depression seems to act more as an independent risk factor to cardiovascular events, rather, than promote them through indirect means [26]. The fact that only one factor confounded the relationship seems to point in this direction. The role that cardiovascular drugsdrug play in this relationship requires further study.

### Strengths and limitations

The longitudinal nature of the data allows insight into the relationship between depression and cardiovascular diseases over time. Due to the relatively short follow-up, the relatively small numbers of participants and the limited numbers of events, we were only able to apply a simple association model. An interesting comparison would be to study whether older patients treated for depression have a better cardiovascular prognosis. More research is needed with longer follow-up time to allow for more accurate estimations of the associations. As our study is one of the few studies that used a general practice population, the factors we studied are relevant. The sampling may partly explain why so many participants had a high CVD-risk at baseline. These were mostly patients in GP care for their chronic conditions. Lack of confirmation of the PRIME-MD diagnosis by clinician interview is a final limitation.

## Conclusions

In conclusion, we found that MDD is a risk factor for CVD in older primary care patients, over and above other well-known cardiovascular risk factors. Within a 2 year follow-up period, there was an almost threefold increase in CVD onset among depressed patients compared with controls.
